# Prospective observational study on behavioral monitoring, disease progression assessment, and screening model development for patients with depression using wearable devices and mobile phones: protocol

**DOI:** 10.3389/fpsyt.2026.1637109

**Published:** 2026-03-23

**Authors:** Mingqia Wang, Fei Peng, Wanrong Mu, Yanbao Tao, Haiya Zhao, Qiuju Yin, Zhijun Yan, Chuan Shi

**Affiliations:** 1 Peking University Sixth Hospital, Peking University Institute of Mental Health, Beijing, China; 2 National Health Commission (NHC) Key Laboratory of Mental Health (Peking University), National Clinical Research Center for Mental Disorders (Peking University Sixth Hospital), Beijing, China; 3 School of Management, Beijing Institute of Technology, Beijing, China

**Keywords:** digital phenotyping, ecological momentary assessment (EMA), major depressive disorder, prospective cohort study, wearable devices

## Abstract

**Background:**

Depression affects over 95 million people in China (lifetime prevalence 6.8%), yet traditional assessments rely on episodic, retrospective evaluations missing dynamic symptom fluctuations. While digital phenotyping offers continuous monitoring potential, feasibility questions remain for psychiatric populations: patient retention, data completeness, and technical challenges. This study assesses the feasibility of intensive multimodal digital phenotyping in routine outpatient care and explores digital phenotypes in major depressive disorder (MDD).

**Methods and analysis:**

This is a prospective protocol for a 12-month observational cohort study at Peking University Sixth Hospital that recruited 202 MDD outpatients and 100 healthy adults (aged 18–60 years) from August 2023 to February 2025, with follow-up ongoing through February 2026. The study evaluates practicality and reliability of multimodal digital phenotyping in real-world clinical settings. Data collection integrates: (1) high-frequency clinician-administered assessments using the 17-item Hamilton Depression Rating Scale (HAMD-17) at seven time points for patients with MDD and at baseline for healthy participants; (2) daily ecological momentary assessments and biweekly self-report questionnaires; (3) passive smartphone sensor data (physical activity, GPS, screen usage*, etc.*); (4) continuous wearable data (sleep, heart rate variability, step count*, etc.*); and (5) environmental exposures (photoperiod, temperature, air pollution*, etc.*) linked to GPS coordinates. Primary outcomes include recruitment and retention rates, data completeness across modalities, and platform-specific constraints. Secondary analyses—prospectively planned pending full data collection—will (1) characterize digital phenotypes and temporal dynamics; (2) compare MDD vs. healthy participants; (3) explore environment-symptom associations.

**Results:**

MDD patients (n=202) were younger (30.8 ± 10.1 vs 42.8 ± 11.4 years) and more often unmarried (42.1% vs 15.0%) than healthy participants (n=100). Clinical-assessment completion declined from 100% (baseline) to 68.3% (Week 8). Among Android users (n=214), adequate EMA completion was 31.6% (MDD) vs 84.0% (healthy). Wearable adherence was 29.2% vs 70.0% (n=302).

**Conclusion:**

This feasibility study reveals marked compliance disparities between MDD patients and healthy participants, highlighting implementation barriers for real-world digital phenotyping in psychiatric populations.

## Introduction

Depression is a major global public health challenge, affecting an estimated 280 million people worldwide and exceeding 95 million individuals in China (1–4). Beyond its high prevalence, Major Depressive Disorder (MDD) is characterized by substantial temporal variability (5), with symptoms fluctuating across hours, days, and seasons (6). Yet, routine clinical care still relies largely on infrequent, clinic-based assessments and retrospective self-report. These low-frequency, recall-based evaluations are poorly suited to capturing rapid symptom changes (7), contextual triggers (8), and individual heterogeneity in illness trajectories, limiting opportunities for early detection of relapse, timely treatment adjustment, and implementation of precision psychiatry approaches (9, 10) that require fine-grained, ecologically valid data on symptom dynamics (11, 12).

Depression arises from complex interactions among biological vulnerabilities, environmental exposures, and behavioral patterns (13). Environmental factors (14) such as photoperiod, temperature, and air pollution, as well as behavioral dimensions including sleep–wake regularity, physical activity (15), and digital device use (16), have all been implicated in the onset and course of depressive symptoms (17–19). However, conventional longitudinal methods—periodic questionnaires, clinic visits, and retrospective diaries—struggle to capture these processes with sufficient temporal resolution, are vulnerable to recall bias, and only partially quantify relevant behaviors and environmental exposures (20, 21). In particular, they rarely integrate objective measures of daily activity, sleep, mobility, and ambient conditions in real-world settings.

Recent advances in digital phenotyping, combining smartphones and wearable sensors, offer a potential solution (16, 22, 23). These tools can passively and repeatedly capture mobility patterns, sleep and activity metrics, cardiovascular parameters, and aspects of digital behavior, while ecological momentary assessment (EMA) enables near real-time reporting of mood and related symptoms (24–27). Yet, important gaps limit the clinical applicability of existing work (28). Most studies have relied on convenience samples rather than clinically diagnosed patients, have followed participants for only a few weeks to months, have calibrated digital signals against sparse clinical assessments, and have rarely incorporated environmental exposure data (29, 30). Critically, the feasibility and operational challenges of implementing intensive, multimodal digital monitoring over extended periods in routine psychiatric outpatient settings—particularly in the context of heterogeneous smartphone platforms (31)—remain insufficiently understood (30, 32).

The present study contributes to addressing these gaps through a 12-month prospective observational cohort of clinically diagnosed MDD outpatients and healthy participants, integrating repeated clinician-rated assessments, multimodal digital monitoring (smartphone-based EMA and passive sensing, wearable-derived physiological data), and standardized environmental exposure measures. The primary objective is to evaluate the feasibility and operational characteristics of implementing such an intensive digital phenotyping protocol in real-world outpatient care, including recruitment and retention over 12 months, adherence and data completeness across modalities, and practical constraints related to device platforms (e.g., Android vs. iOS). Secondary, hypothesis-generating objectives are to: (1) descriptively characterize digital behavioral–physiological phenotypes and their temporal dynamics in MDD; (2) compare key digital features between MDD patients and healthy participants; and (3) explore within-person associations between daily environmental exposures and fluctuations in mood, sleep, and activity. These analyses are intended to provide preliminary insights and inform the design of future, more definitive studies and potential clinical applications.

## Method and analysis

### Study design

This study is a prospective longitudinal observational study comprising two parallel cohorts: an MDD patient cohort (n=202) and a healthy comparison cohort (n=100). It focuses on patients with depression who are treated in outpatient settings; inpatients are excluded because their mobility and daily activities are substantially restricted and differ markedly from those of outpatients. Outpatients with depression and healthy participants are enrolled and followed over time. For the depression outpatient group, we observe the course of illness, including changes in symptom severity, the dynamic characteristics of symptom manifestations, and patterns of disease progression. For the healthy group, we collect digital behavioral and physiological data over the same 12-month period, with clinical assessments conducted only at baseline, to provide a comparative reference. In this study, all treatments, including medications and psychotherapy, are provided as part of routine clinical care at the discretion of the treating clinicians. The study does not introduce or modify any treatment interventions.

### Study setting

The study is being conducted in the psychiatric outpatient department of Peking University Sixth Hospital, a tertiary psychiatric hospital in Beijing, China.

### Participants

For patients with depression, the inclusion criteria are as follows: (1) aged between 18 and 60 years; (2) meeting the diagnostic criteria for MDD according to the Diagnostic and Statistical Manual of Mental Disorders, Fifth Edition (DSM-5), as assessed by the Mini-International Neuropsychiatric Interview (M.I.N.I. 7.0); (3) confirmed to be in a current depressive episode through the M.I.N.I. 7.0; (4) providing written informed consent; (5) having sufficient literacy and smartphone operation skills to complete the study assessments; and (6) a baseline Hamilton Depression Rating Scale-17 (HAMD-17) total score of 14 or higher (33). The exclusion criteria are as follows: (1) pregnant or lactating women; (2) severe or unstable physical illnesses (judged by the investigators to potentially interfere with participation in the study); (3) a history of neurological disorders (such as epilepsy, traumatic brain injury, multiple sclerosis, motor neuron disease, Parkinson’s disease, cerebellar ataxia); and (4) a diagnosis of other severe psychiatric disorders (excluding anxiety disorders) according to the M.I.N.I. 7.0 (34, 35), including organic mental disorders, substance use disorders, schizophrenia spectrum disorders, or bipolar disorder. For healthy individuals, the inclusion criteria are as follows: (1) aged between 18 and 60 years; (2) providing written informed consent; (3) having sufficient literacy and smartphone operation skills to complete the scale assessments; (4) a baseline HAMD-17 (36) total score of less than 14; and (5) using an Android-based smartphone. The exclusion criteria are as follows: (1) pregnant or lactating women; (2) severe or unstable physical illnesses (judged by the investigators to potentially interfere with participation in the study); (3) a personal history of psychiatric disorders or a first-degree family history of psychiatric disorders; (4) any current psychiatric disorder according to the M.I.N.I. 7.0, and (5) a history of traumatic brain injury or other neuropsychiatric disorders.

### Recruitment and follow-up plan

**Figure 1:**
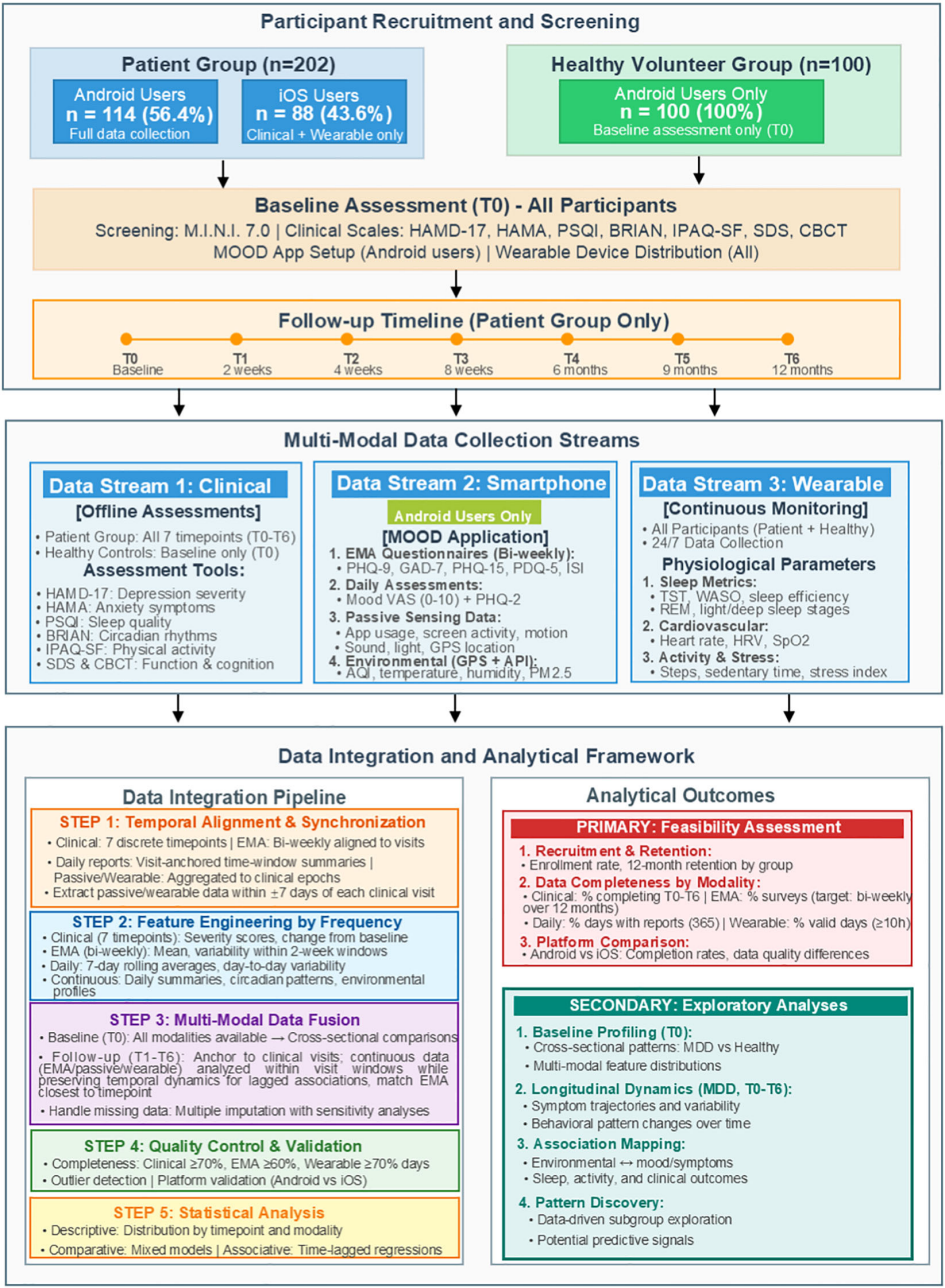
Schematic overview of study design, multi-modal data collection, and analytical framework.

Participant recruitment was completed between August 2023 and February 2025, with 12-month follow-up assessments currently ongoing. Patients with MDD were recruited consecutively from the outpatient psychiatry clinic during routine clinical visits. Healthy participants were recruited concurrently from Beijing residents (with ≥1 year of local residence) through social media advertisements and community-based announcements. All participants must be able to remain in Beijing throughout the 12-month study period to ensure follow-up feasibility. Written informed consent is obtained from all participants prior to enrollment (**Figure 1**).

The study employed a longitudinal assessment protocol with seven evaluation timepoints: baseline (T0), and follow-up assessments at 2 weeks (T1), 4 weeks (T2), 8 weeks (T3), 6 months (T4), 9 months (T5), and 12 months (T6) post-enrollment. Patients with MDD completed clinical and psychometric assessments at all timepoints, while healthy participants completed assessments at baseline (T0) only. The temporal schedule of clinical assessments is detailed in **Table 1**. Data collection is expected to be completed by February 2026.

**Table 1:** Temporal schedule of clinical and psychometric assessments.

Assessment instruments	Baseline assessment (T0)	2-week follow-up (T1)	4-week follow-up (T2)	8-week follow-up (T3)	6-month follow-up (T4)	9-month follow-up (T5)	1-year follow-up (T6)
Eligibility screening	P/H						
Written informed consent	P/H						
Basic information (demographic data, etc.)	P/H						
HAMD-17	P/H	P	P	P	P	P	P
HAMA	P/H	P	P	P	P	P	P
PSQI	P/H	P	P	P	P	P	P
CBCT	P/H			P	P	P	P
BRAIN	P/H	P	P	P	P	P	P
IPAQ-SF	P/H		P	P	P	P	P
SDS	P/H		P	P	P	P	P

P/H, both patients with major depressive disorder and healthy participants; P, patients only.

BRIAN, Biological Rhythms Interview of Assessment in Neuropsychiatry; CBCT, Chinese Cognitive Assessment Scale; HAMA, Hamilton Anxiety Rating Scale; HAMD-17, 17-item Hamilton Depression Rating Scale; IPAQ-SF, International Physical Activity Questionnaire-Short Form; PSQI, Pittsburgh Sleep Quality Index; SDS, Sheehan Disability Scale.

### Data collection

Data collection was organized into three main components based on data source and acquisition device: (1) clinician-administered clinical and psychometric assessments (**Table 1**); (2) Android smartphone–based digital assessments and passive sensing, including GPS-linked environmental exposure (**Table 2**); and (3) wearable monitoring using a Xiaomi smart band (**Table 3**).

**Table 2:** Android smartphone-based digital data (MOOD app).

Data domain	Variables	Source	Mode	Sampling protocol	Derived features
Symptom scales (EMA)	PHQ-9, GAD-7, PHQ-15, PDQ-5, ISI	In-app questionnaires	Active	Bi-week	Scale scores, longitudinal trajectories
Daily mood	VAS (0-10): “Right now, how is your mood?”	In-app single item	Active	Once daily, flexible timing	Daily ratings, variability, trends
Momentary symptoms	PHQ-2 (current state): feeling down, loss of interest	In-app screener	Active	Once daily with VAS	Momentary scores; correlation with VAS/PHQ-9
App usage	Opening/closing events, usage duration	Android UsageStats API	Passive	Continuous event logging	Screen time, app switches, app categories, nocturnal use
Screen activity	On/off, lock/unlock events	Android system	Passive	Continuous event logging	Unlocks per day, session duration, circadian patterns
Environmental exposure	AQI; temperature; humidity; PM2.5; PM10; O_3_; NO_2_; SO_2_	Seniverse API + GPS	Passive	Daily query based on GPS location	Daily queries based on GPS location; matched by date/location
Acoustic environment	Sound intensity (dB)	Microphone	Passive	15-sec segments every 10 min at 10 Hz	Hourly/daily averages, noise exposure
Smartphone-based physical activity	Acceleration, gyroscope, orientation, magnetometer, gravity	Motion sensors	Passive	15-sec bursts every 10 min at 10 Hz	Movement intensity, sedentary/active periods, circadian activity
Light exposure	Illuminance (lux)	Light sensor	Passive	15-sec bursts every 10 min at 10 Hz	Daily mean/max lux, bright light timing, nocturnal exposure

AQI, Air Quality Index; API, Application Programming Interface; dB, decibels; EMA, ecological momentary assessment; GAD-7, Generalized Anxiety Disorder 7-item scale; GPS, Global Positioning System; Hz, Hertz; ISI, Insomnia Severity Index; NO_2_, nitrogen dioxide; O_3_, ozone; PDQ-5, Perceived Deficits Questionnaire 5-item; PHQ-2/9/15, Patient Health Questionnaire 2-/9-/15-item; PM2.5/PM10, particulate matter ≤2.5/10 μm; SO_2_, sulfur dioxide; VAS, Visual Analog Scale.

**Table 3:** Xiaomi mi band 7 pro wearable-derived data.

Data category	Variables	Sensor/method	Sampling	Derived features
Sleep	Total sleep time, WASO, sleep latency, wake time, bedtime, sleep efficiency, light/deep/REM duration	Accelerometer + PPG; proprietary algorithm	Automatic nocturnal monitoring	Nightly metrics, sleep efficiency, weekly averages
Cardiovascular	Resting HR, continuous HR, SpO_2_	PPG sensor (green LED for HR; red/infrared for SpO_2_)	Every ~10 min; minute-level export	Daily resting/mean/max HR, circadian profile, SpO_2_ levels
Physical activity	Step count, energy expenditure, sedentary events (>30 min)	3-axis accelerometer	Continuous monitoring	Daily steps, kcal, sedentary bouts, weekly averages
Stress	Stress index (0–100): relaxed (0–29), mild (30–59), moderate (60–79), high (80–100)	PPG-based HRV; proprietary algorithm	Periodic computation	Daily scores, time in each category
Adherence	Wear time (hrs/day), valid days (≥10 hrs/day)	Accelerometer + PPG continuity	Continuous monitoring	Daily wear time, weekly adherence rate

HR, heart rate; HRV, heart rate variability; LED, light-emitting diode; max, maximum; PPG, photoplethysmography; REM, rapid eye movement sleep; SpO_2_, peripheral capillary oxygen saturation; WASO, wake after sleep onset.

Clinician-administered assessments were conducted during on-site visits at the psychiatric outpatient department. A self-designed demographic questionnaire was used to collect sociodemographic information, including age, gender, ethnicity, education level, marital status, employment status, dietary habits, and reproductive health history. A separate clinical characteristics registry documented body mass index (BMI), medication allergies, age of onset, date of diagnosis, number of prior hospitalizations, current psychotropic medications, tobacco and alcohol use, and physical and psychiatric comorbidities. Standardized clinical and psychometric instruments included the 17-item Hamilton Depression Rating Scale-17 (HAMD-17) (36), Hamilton Anxiety Rating Scale (HAMA) (37), Pittsburgh Sleep Quality Index (PSQI) (38), Biological Rhythms Interview of Assessment in Neuropsychiatry (BRIAN) (39, 40), International Physical Activity Questionnaire-Short Form (IPAQ-SF) (41), Sheehan Disability Scale (SDS) and Chinese Cognitive Assessment Scale (CBCT) (42). These measures were administered face to face by psychiatrists or trained research staff at baseline and scheduled follow-up visits. The timing of each clinician-administered assessment is detailed in **Table 1**.

Smartphone-based data were collected via a dedicated Android application. Only participants owning a compatible Android smartphone and consenting to digital data collection were enrolled in the smartphone-based component. Environmental exposure data were obtained by integrating the Seniverse Weather API (https://www.seniverse.com/api) with GPS-derived geographic coordinates from the participant’s smartphone. The following environmental variables were retrieved: air quality index (AQI), daily average temperature (°C), relative humidity (%RH), fine particulate matter (PM_2.5_, μg/m³; PM_10_, μg/m³), ozone (O_3_, μg/m³), nitrogen dioxide (NO_2_, μg/m³), and sulfur dioxide (SO_2_, μg/m³). Passive sensing captured behavioral and contextual information from the Android device. App usage logs recorded application opening and closing events and usage duration. Screen activity logs included screen-on, unlock, lock, and screen-off events. Microphone data sampled 15-second audio segments every 10 minutes at a frequency of 10Hz, recording sound intensity (decibels, dB). Sensor data were obtained from built-in motion and orientation sensors of the Android smartphone (including accelerometers, gyroscopes, orientation sensors, magnetometers, gravity sensors, linear acceleration sensors, and light sensors), sampled in 15-second windows every 10 minutes at 10 Hz. Android smartphone–based active self-reports were used for high-frequency symptom monitoring between clinic visits. At predefined app-based assessment timepoints, participants completed the Patient Health Questionnaire-9 (PHQ-9) (43), Generalized Anxiety Disorder Scale-7 (GAD-7) (44), Patient Health Questionnaire-15 (PHQ-15) (45), Perceived Deficits Questionnaire-5 (PDQ-5) (46), and Insomnia Severity Index (ISI). For daily dynamic monitoring, participants were asked to complete a brief mood and symptom check once per day via the smartphone application. To maximize ecological validity and long-term adherence, no fixed assessment time was imposed; instead, participants received one daily reminder (8 am) and could choose a convenient moment during the day to respond. At each assessment, mood state was measured using a 0–10 Visual Analog Scale (VAS) (47) with the prompt “Right now, how is your mood?”(0 = very bad, 10 = very good). In addition, a modified momentary version of the two-item Patient Health Questionnaire depression screener (PHQ-2) (48) was administered, in which the original 2-week time frame was adapted to assess current state (e.g., “Right now, how much are you bothered by feeling down, depressed, or hopeless?”). All responses were time-stamped as part of the digital log.

Wearable device monitoring was conducted using Xiaomi Mi Band 7 Pro smart bands (Xiaomi Corporation, Beijing, China) for continuous biometric and behavioral tracking throughout the 12-month study period. All participants were provided with a study device at enrollment and instructed to wear it continuously, including during sleep, except when charging or during water-related activities that might compromise device function. The bands recorded four main categories of data. First, sleep parameters (49) included total sleep time (TST, hours), wake after sleep onset (WASO, minutes), sleep onset latency (minutes), wake-up time, bedtime, sleep efficiency (%), and sleep architecture (duration of light, deep, and REM sleep stages in minutes). These metrics were automatically derived by the device’s proprietary sleep-detection algorithm, which integrates triaxial accelerometry and photoplethysmography (PPG)-based heart rate signals to identify sleep-wake transitions and classify sleep stages. Second, cardiovascular and autonomic parameters comprised resting heart rate (beats per minute, measured during sleep and prolonged sedentary periods), continuous heart rate monitoring throughout wear time, and peripheral oxygen saturation (SpO_2_, %, measured via a dual-wavelength optical sensor). Cardiovascular signals were sampled continuously or at regular intervals (approximately every 10 minutes) using the built-in PPG sensor, and minute-level summaries were exported via the Xiaomi Health mobile application. Third, physical activity and sedentary behavior (50) included daily step count, estimated energy expenditure (kcal), and sedentary events, defined as continuous sedentary periods exceeding 30 minutes as identified by sustained low movement on the accelerometer, with all metrics derived from triaxial accelerometer data processed using Xiaomi’s proprietary algorithms. Fourth, the device provided a proprietary stress index calculated from heart rate variability and heart rate patterns as an indicator of autonomic nervous system activity and perceived stress. Stress scores ranged from 0 to 100 and were categorized by the vendor as relaxed (0–29), mild (30–59), moderate (60–79), and high (80–100); daily stress scores and the distribution of time spent in each stress category were extracted from the Xiaomi Health app. All wearable data were synchronized to participants’ smartphones via Bluetooth and uploaded to the Xiaomi Health cloud platform, from which they were retrieved by the research team through authorized API access or manual export in standardized formats (e.g., CSV). Device wear time (hours per day) and the number of valid wear days (defined as ≥10 hours of continuous monitoring per day) were calculated as adherence and data-quality indicators and used in subsequent analyses. The Xiaomi Mi Band 7 Pro is equipped with a 3-axis accelerometer, a PPG optical heart rate sensor (green LED for heart rate; dual red/infrared LEDs for SpO_2_), an ambient light sensor, and onboard data-processing capabilities, and provides a typical battery life of approximately 10–12 days, thereby minimizing charging-related gaps in data collection.

### Smartphone operating system and digital data collection

The study’s custom digital phenotyping application (MOOD app) was developed exclusively for Android devices due to technical and operating system–level constraints on data access in iOS. For patients with MDD, smartphone operating system was not used as an inclusion or exclusion criterion. Patients using Android devices (n = 114, 56.4%) contributed the full suite of digital data (passive smartphone sensing, EMA, GPS-based environmental exposures, and wearable monitoring), whereas patients using iOS devices (n = 88, 43.6%) contributed clinical assessments and wearable-derived data only. Healthy participants were required to use Android smartphones so that complete digital reference data could be obtained.

### Quality control

Quality control was ensured through an integrated, multimodal data collection and management system. A bidirectional data flow between participants’ devices and the central data management team supported continuous monitoring and feedback. Data were obtained through three main modalities: (1) Clinical assessments: Paper-based instruments administered by trained, certified clinicians during face-to-face evaluations, with subsequent dual independent data entry into electronic case report forms (eCRFs) using EpiData software (version 3.1), maintaining an error rate below 0.2%. (2) Physiological monitoring: Continuous physiological signals (heart rate, step count, sleep patterns, activity levels) collected via Xiaomi Mi Band 7 wristbands and automatically synchronized through the Xiaomi Health mobile application. (3) Digital behavioral data: EMA responses and passive smartphone usage patterns captured by a custom Android application developed by the Beijing Institute of Technology (BIT).

### Data security architecture

A structured, multi-tier storage approach ensured compliance with regulatory requirements and maintains scientific integrity. Real-time data collected via mobile devices——including EMA responses, smartphone sensor data, and wearable device metrics——were encrypted using AES-256 algorithms and stored on secure cloud servers during the active data collection phase. Upon study completion, the complete dataset will be transferred to secure, offline local servers at Peking University Sixth Hospital (PKUH6) for long-term storage and analysis. In accordance with Chinese national standards (GB/T series), all paper-based clinical health records were stored in access-controlled, locked cabinets and retained for a minimum of five years. Access to both digital and physical records was restricted to authorized research personnel only, with all data handling procedures logged and audited regularly.

### Phased quality assurance protocol

Quality assurance is implemented across three distinct phases to ensure data reliability and scientific rigor. Pre-implementation phase: Protocol standardization was achieved through the development and implementation of a Research Operations Manual (version 2.3). All clinical assessors completed mandatory training and certification, achieving inter-rater reliability (Cohen’s κ ≥ 0.85) for MINI 7.0 diagnostic interviews. Wearable devices and smartphones underwent technical calibration to ensure measurement consistency across the study cohort. Active monitoring phase: Real-time compliance monitoring combined automated systems and human oversight. Participants received daily push notifications (8:00 AM) for the first 8 weeks for daily mood assessments. After week 8, cascading SMS reminder notifications were sent within a 3-day window (± 3 days) surrounding biweekly EMA assessment dates to maximize response rates. Automated algorithms flagged potential data quality issues, including wearable device discontinuities exceeding 7 consecutive days. Research staff maintained consistent participant-assessor pairings throughout the study period to minimize inter-rater variability. Post-collection phase: A three-tier data validation workflow is implemented using EpiData’s verification engine. First, automated algorithms flag anomalies based on pre-defined logical checks (e.g., out-of-range values, temporal inconsistencies). Second, trained data managers review flagged entries against source documentation. Third, senior data administrators adjudicate unresolved discrepancies. Prior to formal statistical analysis, processing pipelines are validated using synthetic datasets that simulate the structure and complexity of real study data.

### Analysis plan

Feasibility outcomes will be evaluated using descriptive statistics and reported with 95% confidence intervals where appropriate. We will estimate the recruitment success rate as the proportion of approached eligible patients who provide consent. Among enrolled participants, we will examine 8-week and 12-month retention rates stratified by baseline demographic and clinical characteristics, and characterize time-to-dropout patterns using Kaplan-Meier survival curves. For each data modality (EMA, passive sensing, wearables, clinical assessments), we will quantify the proportion of participants achieving adequate compliance thresholds [e.g., ≥70% for EMA and passive sensing data (42); ≥4 days/week for wearables (51)], mean and median compliance rates over time, and temporal adherence patterns including decay over time and weekend effects. Missing data patterns will be characterized by proportion and duration of missing episodes, temporal distribution across study phases and days of the week, participant-level factors associated with low compliance or data loss (including demographic, clinical, and platform-related variables such as Android vs. iOS), and the impact of platform constraints on multimodal data availability.

All secondary analyses are hypothesis-generating and will be conducted on participants with adequate data completeness. For MDD patients with complete multimodal data, we will systematically describe temporal patterns and inter-individual variability in key behavioral-physiological domains using visualization methods and summary statistics, including sleep parameters (duration, efficiency, timing, regularity), physical activity patterns (step counts, sedentary time, activity rhythms), cardiovascular dynamics (resting heart rate, heart rate variability), smartphone usage behaviors (screen time, app categories, diurnal patterns), and real-time mood states from EMA, with particular emphasis on the acute treatment phase (baseline to 8 weeks), when data availability and sample size are expected to be highest. Between-group comparisons will be conducted to examine differences in digital phenotypic features between MDD patients and healthy participants using appropriate statistical tests (t-tests, Mann-Whitney U tests, or linear regression models adjusted for demographic covariates including age, sex, education, and socioeconomic status), with effect sizes reported with confidence intervals; given the exploratory nature, we will not apply strict multiple comparison corrections but will interpret findings in the context of the number of tests performed. For participants with sufficient longitudinal data, linear mixed-effects models will be used to examine temporal trajectories of digital markers during treatment (with random intercepts and slopes to account for inter-individual variability) and associations between daily environmental exposures (photoperiod, ambient temperature, air quality [PM2.5]*, etc*) and same-day or next-day fluctuations in mood, activity, and sleep, modeled as within-person effects with appropriate lag structures and adjusted for relevant time-varying and time-invariant covariates; seasonal effects will be explored where longitudinal coverage permits.

### Analytical progress to date

Preliminary analyses have been conducted to evaluate feasibility, compliance, and clinical outcomes. Between-group comparisons of sociodemographic and clinical variables were performed using independent samples t-tests for continuous measures and chi-square tests for categorical variables. Standardized mean differences (SMD) were calculated to quantify effect sizes. Data completeness was quantified across three modalities: (1) clinical assessment retention rates with 95% confidence intervals calculated using the Wilson score method; (2) EMA completion rates with ≥70% defined as adequate compliance (42) and (3) wearable device wear compliance with adequate compliance operationalized as ≥4 days/week, ≥10 hours/day wear time (51). Within-group changes in HAMD-17 and HAMA scores from baseline to weeks 2, 4, and 8 were evaluated using paired t-tests at each timepoint. Cohen’s d effect sizes with 95% confidence intervals were computed to quantify clinical significance of symptom change. All analyses were conducted in R version 4.5.1, with statistical significance set at 0.05 (two-tailed). Missing data were handled using available case analysis, with sample sizes reported at each timepoint.

### Missing value handling

We will prioritize conducting analyses using available data without filling in missing values when dealing with data from clinician-administered face-to-face assessments and EMA data. Additionally, we intend to conduct Full Analysis Set (FAS) and Per-Protocol Set (PPS) analyses. In addition to these methods, we will also employ multiple imputation to handle the data comprehensively. Regarding passive behavior monitoring data, we will record the timestamps of collected behavioral events without filling in missing values to preserve the original distribution of the data. For objective environmental data like weather, we will either get and fill the data within a specific time frame based on the participants’ latitude and longitude coordinates or keep the incomplete information empty for comparison analysis.

### Sample size and rationale

As the primary aim of this study is to assess feasibility and operational characteristics of intensive multimodal digital phenotyping, the target sample size was determined based on precision requirements for estimating key feasibility parameters rather than power calculations for effect size detection (52, 53). We aim to recruit 200 MDD patients, justified by the following considerations. First, using confidence interval–based approaches recommended for feasibility and pilot studies, a sample of 200 MDD patients provides an approximately 95% confidence interval margin of error of ±7 percentage points when estimating a proportion around 50% [e.g., recruitment success, retention, or the proportion achieving adequate EMA adherence ≥70% daily compliance), which was judged to offer adequate precision for feasibility assessment. Second, given that MOOD app data collection is technically feasible only on Android devices, we will implement platform-aware recruitment targeting approximately 150 Android users and 50 iOS users (reflecting the ~75:25 distribution in the Chinese smartphone market (54)]. Accounting for anticipated 20-25% attrition over the 8-week intensive monitoring period and 80% data completeness among retained participants, this approach is expected to yield approximately 90–100 participants with complete multimodal sensing data for secondary exploratory analyses, with an estimated 55–60 participants retained for extended 12-month longitudinal modeling. A target of 100 healthy participants provides adequate reference data for descriptive characterization and basic between-group comparisons.

## Results

**Figure 2:**
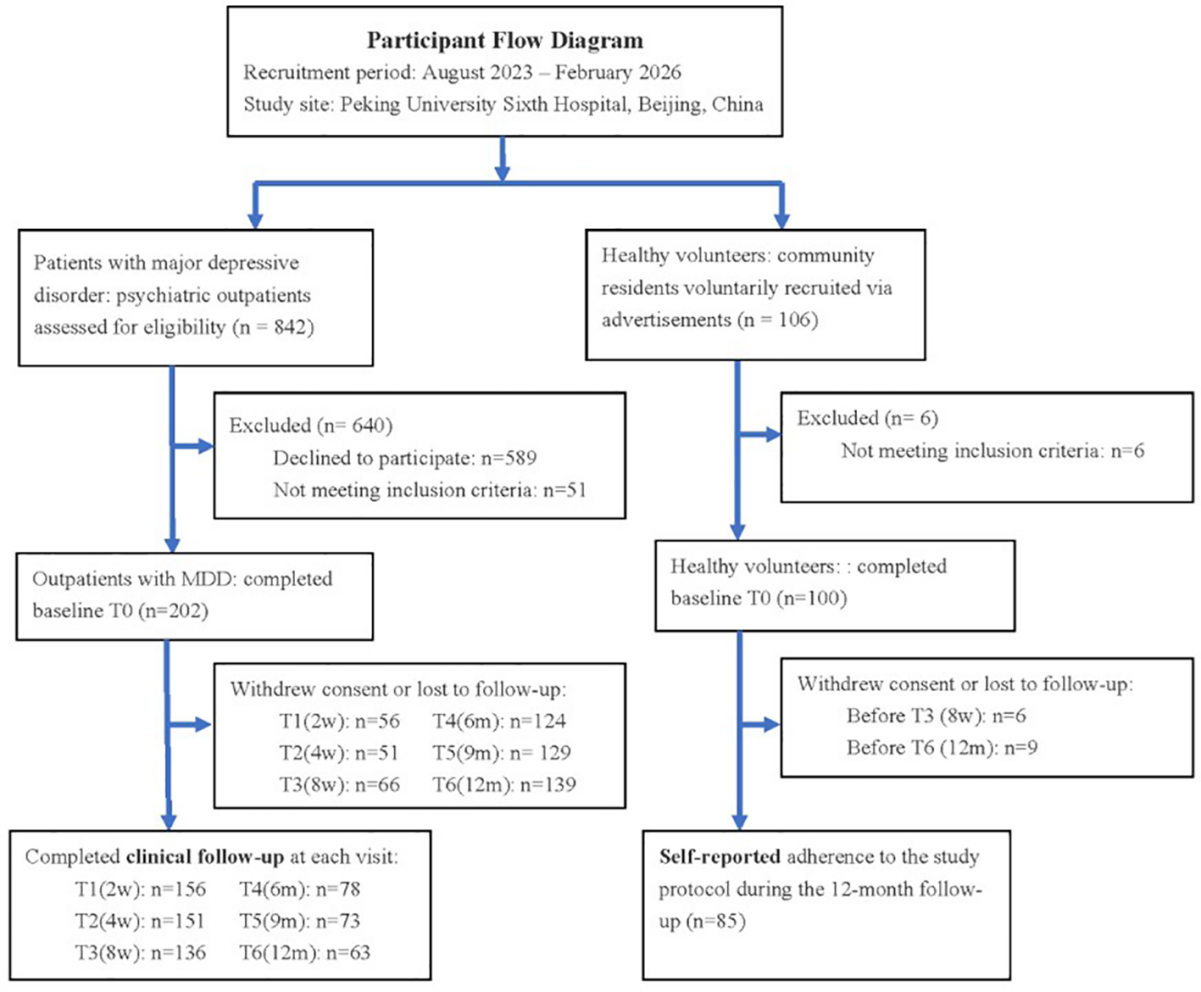
Participant flow from screening through 12-month follow-up.

**Figure 2** shows the participant flow through screening, enrollment, and clinical follow-up. Baseline characteristics are presented in **Table 4**. MDD patients were significantly younger than healthy participants (30.77 ± 10.08 vs 42.80 ± 11.35 years; *t* = -8.99, *p*<0.001, SMD = 1.121), but sociodemographic confounders including sex (*p* = 0.474), education (*p* = 0.283), employment (*p* = 0.25), and BMI (*p* = 0.101) were well-balanced (all SMD <0.2). MDD patients showed higher unmarried rate (42.1% vs 15.0%; χ² = 20.94, *p*<0.001). Depression and anxiety severity differed substantially between groups (HAMD-17 *t* = 47.55, HAMA *t* = 38.63; both *p*<0.001), confirming valid case-control differentiation. Past medical history prevalence was similar (28.7% vs 27.0%; χ² = 0.03, *p* = 0.861). Across the 8-week study, clinical assessment completion declined from 100% at baseline (202/202) to 77.2% at Week 2, 74.8% at Week 4, and 68.3% at Week 8 in MDDs (**Table 5A**). For daily EMA completion among Android users (n=214), adequate compliance (≥70% of prompted surveys) was achieved by 56.1%, with marked group differences: 84.0% in healthy participants versus 31.6% in MDD (**Table 5B**). Low EMA completion (1–49%) was uncommon in healthy participants (2.0%) but frequent in MDD (52.6%). Wearable adherence showed a similar pattern (n=302): meeting the predefined threshold (≥4 days/week and ≥10 hours/day) was observed in 42.7% overall, including 70.0% of healthy participants and 29.2% of MDD; notably, 59.9% of MDD participants had only 0–20 valid wear days (**Table 5C**). Longitudinally, patients demonstrated substantial symptom improvement. HAMD-17 decreased from 21.22 at baseline to 10.60 at Week 8 (mean change −10.75; Cohen’s *d*=1.62), and HAMA decreased from 21.63 to 11.42 (mean change −10.36; *d*=1.35), indicating sustained reductions in depressive and anxiety symptoms over follow-up (**Table 6**).

**Table 4:** Baseline sociodemographic and clinical characteristics of study participants.

Demographic characteristics	MDD patients (N = 202)	Healthy participants (N = 100)	Statistic	p-value	SMD
Age, years (mean ± SD)	30.77 ± 10.08	42.80 ± 11.35	-8.99	<0.001	1.121
Age ≤35 years, n (%)	139 (68.8)	28 (28.0)	43.43	<0.001	0.895
Sex (Female), n (%)	143 (70.8)	66 (66.0)	0.51	0.474	0.103
Years of Education (mean ± SD)	13.83 ± 3.50	14.29 ± 3.28	-1.10	0.283	0.134
Education Level, n (%)			0.22	0.878	0.057
Secondary School or Below(7–12 years)	65 (32.3)	34 (34.7)			
University (13–16 years)	104 (51.7)	48 (49.0)			
Postgraduate (>16 years)	32 (15.9)	16 (16.0)			
Employment Status (Working), n (%)	137 (67.8)	75 (75.0)	1.32	0.25	0.159
Marital Status (Unmarried), n (%)	85 (42.1)	15 (15.0)	20.94	<0.001	0.629
Living Arrangement (with others), n (%)	163 (81.1)	89 (89.0)	3.20	0.074	0.252
Smoking Status (Yes), n (%)	47 (23.4)	14 (14.0)	2.95	0.086	0.238
Alcohol Use (Yes), n (%)	39 (19.5)	10 (10.0)	3.61	0.057	0.267
Body Mass Index, kg/m² (mean ± SD)	22.85 ± 5.13	25.87 ± 24.67	-1.21	0.101	0.17
HAMD-17 Score (mean ± SD)	21.22 ± 5.06	1.34 ± 2.19	47.55	<0.001	5.099
HAMA Score (mean ± SD)	21.63 ± 7.40	0.46 ± 1.67	38.63	<0.001	3.946
Past Medical History of Physical Disease, n (%)	58 (28.7)	27 (27.0)	0.03	0.861	0.038
History of Diabetes, n (%)	3 (1.6)	1 (1.0)			
History of Hypertension, n (%)	6 (3.3)	8 (8.0)			
History of Hyperlipidemia, n (%)	4 (2.2)	8 (8.0)			
History of Coronary Heart Disease, n (%)	1 (0.5)	0 (0.0)			
History of Stroke, n (%)	1 (0.5)	1 (1.0)			
History of Head Trauma, n (%)	2 (1.1)	0 (0.0)			
History of Thyroid Disease, n (%)	4 (2.2)	0 (0.0)			
History of Epilepsy, n (%)	0 (0.0)	0 (0.0)			
History of Transient Ischemic Attack, n (%)	0 (0.0)	0 (0.0)			
History of Surgical History, n (%)	43 (23.5)	17 (17.0)			
History of Other Diseases, n (%)	15 (8.3)	1 (1.0)			

HAMA, 14-item Hamilton Anxiety Rating Scale; HAMD-17, 17-item Hamilton Depression Rating Scale; kg/m², kilograms per meter squared; MDD, major depressive disorder; n, number of participants in subgroup; N, total number of participants; SD, standard deviation; SMD, standardized mean difference.

**Table 5A:** Clinical assessment completion among patients (n = 202) from baseline to week 8.

Timepoint	Completion (n/N)	Completion rate	95% CI
Baseline (T0)	202/202	100.0%	
2-Week Follow-up (T1)	156/202	77.2%	71.0%-82.5%
4-Week Follow-up (T2)	151/202	74.8%	68.3%-80.2%
8-Week Follow-up (T3)	136/202	68.3%	60.6%-73.4%

n, number of patients who completed assessment; N, total number of patients; CI, confidence interval.

**Table 5B:** Daily ecological momentary assessment (EMA) completion by group from baseline to week 8 (Android users only; n = 214).

Completion rate category	HC (n=100)	HC %	MDD (n=114)	MDD %	Total (n=214)	Total %
≥90%	67	67.0%	12	10.5%	79	36.9%
80-89%	10	10.0%	14	12.3%	24	11.2%
70-79%	7	7.0%	10	8.8%	17	7.94%
50-69%	11	11.0%	21	18.4%	32	15.0%
1-49%	2	2.0%	60	52.6%	62	29.0%
≥70% Subtotal	84	84.0%	36	31.6%	120	56.1%

Adequate compliance defined as ≥70% completion rate of prompted daily surveys. The ≥70% subtotal represents participants meeting this compliance threshold.

HC, healthy participants; MDD, patients.

**Table 5C:** Wearable wear compliance by group from the baseline to week 8 (all users with wearable data; n = 302).

Valid days	HC (n=100)	HC %	MDD (n=202)	MDD %	Total (n=302)	Total %
51-56	54	54.0%	21	10.4%	75	24.8%
41-50	10	10.0%	14	6.93%	24	7.95%
31-40	6	6.0%	26	12.9%	32	10.6%
21-30	3	3.0%	20	9.90%	23	7.62%
0-20	27	27.0%	121	59.9%	148	49.0%
≥4 days/week, ≥10 hours/day	70	70.0%	59	29.2%	129	42.7%

Adequate wearable compliance was defined as ≥4 days/week, ≥10 hours/day wear time.

HC, healthy participants; MDD, patients.

**Table 6:** Longitudinal symptom reduction: HAMD-17 and HAMA scores from baseline to week 8 among patients.

Clinical measure	Timepoint	N	Baseline (mean ± SD)	Follow-up (mean ± SD)	Change (mean ± SD)	SE	Cohen’s *d*	95% CI for Cohen’s *d*
HAMD-17 Score	Baseline	202	21.22 ± 5.06	–	–	–	–	–
	Week 2	156	21.15 ± 5.03	14.30 ± 6.80	6.85 ± 5.69	0.456	1.2	0.98–1.43
	Week 4	151	21.04 ± 4.96	12.44 ± 6.74	8.60 ± 5.69	0.463	1.51	1.28–1.74
	Week 8	136	21.35 ± 5.11	10.60 ± 6.66	10.75 ± 6.66	0.571	1.62	1.38–1.85
HAMA Score	Baseline	202	21.63 ± 7.40	–	–	–	–	–
	Week 2	156	21.51 ± 7.01	15.38 ± 8.11	6.13 ± 6.54	0.523	0.94	0.72–1.16
	Week 4	151	21.30 ± 6.92	12.82 ± 7.44	8.48 ± 6.38	0.519	1.33	1.10–1.56
	Week 8	136	21.78 ± 7.40	11.42 ± 7.98	10.36 ± 7.68	0.659	1.35	1.11–1.57

HAMA, Hamilton Anxiety Rating Scale 14-item version; HAMD-17, 17-item Hamilton Depression Rating Scale; N, sample size; SD, standard deviation; SE, standard error.

## Discussion

This study represents a comprehensive effort to implement long-term, multimodal digital phenotyping in Chinese patients with MDD within a real-world clinical setting. As a feasibility-focused investigation, our preliminary recruitment and retention data provide critical insights into the practical challenges and opportunities of deploying intensive digital monitoring protocols in psychiatric populations, with important implications for designing future large-scale studies.

Our recruitment experience reveals substantial barriers to participant engagement in digital phenotyping research. Of 842 outpatients assessed for eligibility, 202 (24%) enrolled and completed baseline assessments, indicating substantial non-participation during recruitment. This high refusal rate likely reflects multiple factors, including concerns about privacy and data security, the perceived burden of continuous smartphone-based monitoring and daily EMAs, technological literacy barriers, and potential apprehension about sharing sensitive behavioral data. These findings underscore the importance of addressing patient concerns through transparent communication about data protection, minimizing participant burden through user-friendly interfaces, and developing culturally sensitive engagement strategies tailored to Chinese clinical populations.

Baseline comparisons further revealed demographic imbalances that warrant careful consideration in future analyses. MDD patients were significantly younger than healthy participants (30.77 vs 42.80 years; SMD = 1.121), potentially reflecting differential clinic attendance patterns, recruitment channel effects, or age-related variations in technology acceptance. While most sociodemographic variables (sex, education, employment, BMI) were well-balanced (all SMD<0.2), the substantial age difference—coupled with higher unmarried rates in the MDD group (42.1% vs 15.0%)—may introduce confounding in digital behavioral features such as smartphone usage intensity, mobility patterns, and sleep-wake rhythmicity. Consequently, exploratory analyses examining digital phenotypes and environment-symptom associations should incorporate age and marital status as covariates or employ stratified/sensitivity analyses to mitigate selection bias and residual confounding.

Early retention data highlight the challenges of maintaining long-term engagement: by 8 weeks, nearly one-third of enrolled patients (31.7%) had withdrawn or were lost to follow-up, with the steepest attrition during the first month (22.8% by Week 2, 25.2% by Week 4). Compliance metrics revealed an even more pronounced group disparity. Among Android users (n=214), adequate EMA completion (≥70% of prompted surveys) was achieved by 84.0% of healthy participants but only 31.6% of MDD patients, with over half of MDD participants (52.6%) completing fewer than 49% of daily assessments. Similarly, wearable adherence (≥4 days/week, ≥10 hours/day) was met by 70.0% of healthy participants versus just 29.2% of MDD patients, with 59.9% of MDD participants accumulating only 0–20 valid wear days. This systematic “compliance gap” likely stems from depression-related motivational deficits, anhedonia, impaired attention and executive function, heightened early-treatment burden, and privacy concerns (55, 56). Reliance on fixed compliance thresholds may systematically underestimate data availability among individuals with greater symptom severity or functional impairment. The acute treatment phase, when symptom burden peaks and patients are adjusting to treatment regimens, presents particular challenges for sustaining compliance with intensive digital monitoring protocols. Future studies should explore adaptive strategies to enhance retention and data completeness, including reduced task burden (shorter instruments or adaptive sampling schedules), personalized reminder systems with motivational feedback, improved device usability (e.g., charging reminders and wear-time coaching), modest incentive structures, and integration with clinical care workflows to reinforce engagement.

An additional operational challenge emerged in the distribution of mobile operating systems among enrolled participants. While we anticipated a relatively balanced distribution based on national smartphone market trends, 56.4% of patients used Android devices compared to 43.6% using iOS. This deviation has important implications for passive sensing data availability, as iOS imposes stricter background data collection restrictions compared to Android, potentially limiting the capture of location patterns, ambient audio features, and continuous activity data on a substantial proportion of participants. This platform-specific constraint necessitates careful consideration in study design, including stratification by operating system during recruitment, development of platform-agnostic data collection protocols where feasible, or analytical approaches that account for differential data completeness across platforms. Future digital phenotyping studies should explicitly incorporate operating system distribution as a feasibility consideration and explore partnerships with device manufacturers or platform-specific technical solutions to maximize data capture uniformity.

Importantly, despite these compliance challenges, enrolled patients demonstrated substantial symptom improvement over the 8-week observation period. HAMD-17 scores declined from 21.22 at baseline to 10.60 at Week 8, and HAMA scores decreased from 21.63 to 11.42, reflecting large effect sizes. These robust trajectories indicate that the cohort exhibited clinically meaningful symptom reduction during the acute treatment phase, providing a sensitive context for capturing digital markers associated with therapeutic response. However, as this was a naturalistic observational study without randomized intervention, symptom improvement should not be interpreted causally, and any identified digital biomarker associations must be regarded as hypothesis-generating and require validation in independent samples. Understanding the factors associated with dropout—including baseline symptom severity, treatment response kinetics, technological barriers, and demographic characteristics—will be essential for developing targeted retention strategies and interpreting digital phenotyping findings in the context of real-world psychiatric care.

Several limitations warrant consideration. First, generalizability may be constrained by the single-center design and probable selection bias toward technology-engaged, urban individuals, with rural populations and severe depression cases underrepresented. Second, smartphone operating system heterogeneity creates significant data completeness challenges: approximately 43.6% of MDD patients used iOS devices that could not contribute passive sensing data due to platform restrictions, while healthy participants were required to use Android devices, potentially introducing selection bias as platform choice correlates with sociodemographic factors in China. Residual confounding by unmeasured characteristics cannot be definitively excluded, and smartphone-derived findings should be interpreted as specific to Android users and require replication in device-unrestricted samples. Third, GPS data quality may be affected by heterogeneous user settings and urban environmental factors (high-rise buildings, underground spaces) that influence signal accuracy. Fourth, while major atmospheric factors are captured, indoor microenvironments (lighting, workplace conditions) are not fully quantified, limiting comprehensive environmental exposure assessment. Fifth, single daily user-initiated momentary mood reports (assessing “right now” rather than the full previous day) prioritize feasibility but introduce timing variability and may not fully represent daily experiences, though this approach is widely used to balance data richness with participant burden in long-term studies (32, 57, 58). Sixth, the 12-month duration may be insufficient to characterize multi-year depression cycles, and once-daily EMA may miss brief critical symptom transitions. Finally, as a feasibility study, all secondary findings should be interpreted as hypothesis-generating and require validation in independent samples.

Despite these challenges and limitations, this study demonstrates the potential feasibility of implementing multimodal digital phenotyping in a Chinese psychiatric clinical setting and positions us to generate important hypothesis-generating findings. If adequate data completeness is achieved, analyses integrating EMAs, passive smartphone sensing, wearable-derived physiological metrics, and clinician-administered assessments may reveal novel insights into the temporal dynamics of depressive symptoms, behavioral patterns, and environmental exposures in real-world contexts. Exploratory examination of associations between photoperiod variability, ambient temperature, air quality, and daily fluctuations in mood and activity could provide preliminary evidence for environmental influences on depression trajectories, potentially informing personalized intervention strategies that account for seasonal and meteorological factors. The 12-month longitudinal design, if retention permits, may capture previously undocumented patterns of symptom recurrence, circadian rhythm disruptions, and long-term treatment response that are difficult to assess through conventional episodic clinical evaluations. These exploratory findings, while requiring validation in independent samples, could collectively inform the development of real-time digital monitoring systems that complement traditional assessment paradigms and enable more responsive, personalized psychiatric care.
